# Secondary open structural rhinoplasty with costal cartilage grafts

**DOI:** 10.1186/s40902-025-00472-x

**Published:** 2025-07-22

**Authors:** Arif Tri Prasetyo, Ali Sundoro, Valdi Muharram Kusumadiningrat

**Affiliations:** 1 Division of Plastic Reconstructive and Aesthetic Surgery, Department of Surgery, Faculty of Medicine, Universitas Padjadjaran, Bandung, Indonesia; 2 Department of Plastic Reconstructive and Aesthetic Surgery, Universitas Padjadjaran Hospital, Sumedang, Indonesia; 3 Division of Plastic Reconstructive and Aesthetic Surgery, Department of Surgery, Dr. Hasan Sadikin General Hospital, Bandung, Indonesia, Bandung, Indonesia

**Keywords:** Cleft lip, Cleft palate, Nasal deformity, Rhinoplasty, Nasal reconstruction, Rib cartilage, Costal cartilage

## Abstract

**Background:**

Cleft lip and palate (CLP) represent one of the most prevalent congenital anomalies of the maxillofacial region, resulting in significant structural deformities that impact nasal function, facial aesthetics, and psychosocial well-being. These anomalies often lead to nasal obstruction and asymmetry. Surgical correction of the associated nasal deformities through rhinoplasty is essential to restore nasal airway function, achieve symmetrical nasal contour, and improve overall facial harmony. Autologous costal cartilage serves as an excellent grafting material due to its versatility, biocompatibility, and structural integrity, making it suitable for reconstructing various components of the nasal framework.

**Case report:**

A retrospective, single-center, non-consecutive case series was conducted at our institution, involving eight patients diagnosed with cleft lip and palate (CLP) between 2019 and 2024. The cohort comprised four male and four female patients, of whom seven presented with unilateral CLP. The mean age at the time of surgery was 21 years. All patients were followed for a duration of 6 months postoperatively. Postoperative assessments demonstrated an increase in the tip projection ratio and a decrease in the alar width ratio, indicating improvement in nasal symmetry and projection.

**Conclusion:**

Secondary structural rhinoplasty using autologous rib cartilage graft remains the definitive surgical approach for correcting nasal deformities in patients with cleft lip and palate (CLP). Despite its effectiveness, the procedure is technically demanding and requires meticulous planning and execution. A systematic and well-considered surgical strategy is essential to achieve optimal nasal tip definition and improved projection. Postoperative evaluations in our series demonstrated significant aesthetic improvements, including enhanced nasal tip projection and a reduction in alar base width, contributing to better nasal symmetry.

## Introduction

Cleft lip and palate (CLP) are among the most prevalent congenital anomalies of the maxillofacial region, characterized by structural deformities that significantly impair oral function, facial aesthetics, and psychosocial well-being from childhood through adulthood [1, 2]. The global prevalence of CLP is estimated at approximately 1 in every 750 live births [3]. The etiology of CLP is multifactorial, involving genetic predisposition, environmental exposures, and socioeconomic factors [4]. Surgical reconstruction of the lip and palate is essential to restore function and improve quality of life. Among the commonly adopted techniques for primary cleft lip repair are the Millard rotation-advancement method and the Tennison-Randall triangular flap [5, 6].

Millard observed that many patients required a secondary surgical intervention following initial cleft repair to optimize functional and aesthetic outcomes [6]. In line with this, Cohen et al. [7] emphasized that no single-stage surgical technique can consistently achieve satisfactory long-term results, underscoring the need for staged, individualized approaches in cleft management.

Cleft nasal (CN) deformity represents one of the most complex and severe nasal abnormalities encountered in clinical practice. Both unilateral and bilateral forms of cleft lip and palate are frequently associated with significant nasal deformities. Typical features include a shortened and deviated columella, asymmetric nasal tip and nostrils, flattened alar cartilages, and a depressed alar base [8]. Nasal septal deviation is commonly observed in individuals with unilateral CLP, often contributing to varying degrees of nasal airway obstruction. Consequently, patients frequently experience impaired nasal breathing, which may predispose them to chronic rhinosinusitis due to altered nasal physiology [9].

Nasal deformities in cleft lip and palate (CLP) patients often become more apparent following primary surgical correction. Secondary reconstruction of the cleft nasal deformity—encompassing the skin, cartilaginous framework, vestibular lining, and underlying bony base—remains a significant challenge for reconstructive surgeons. This complexity arises from several factors, including congenital absence or malposition of nasal structures, variable individual anatomical development, and scarring from previous surgical interventions [10].

**Fig. 1:**
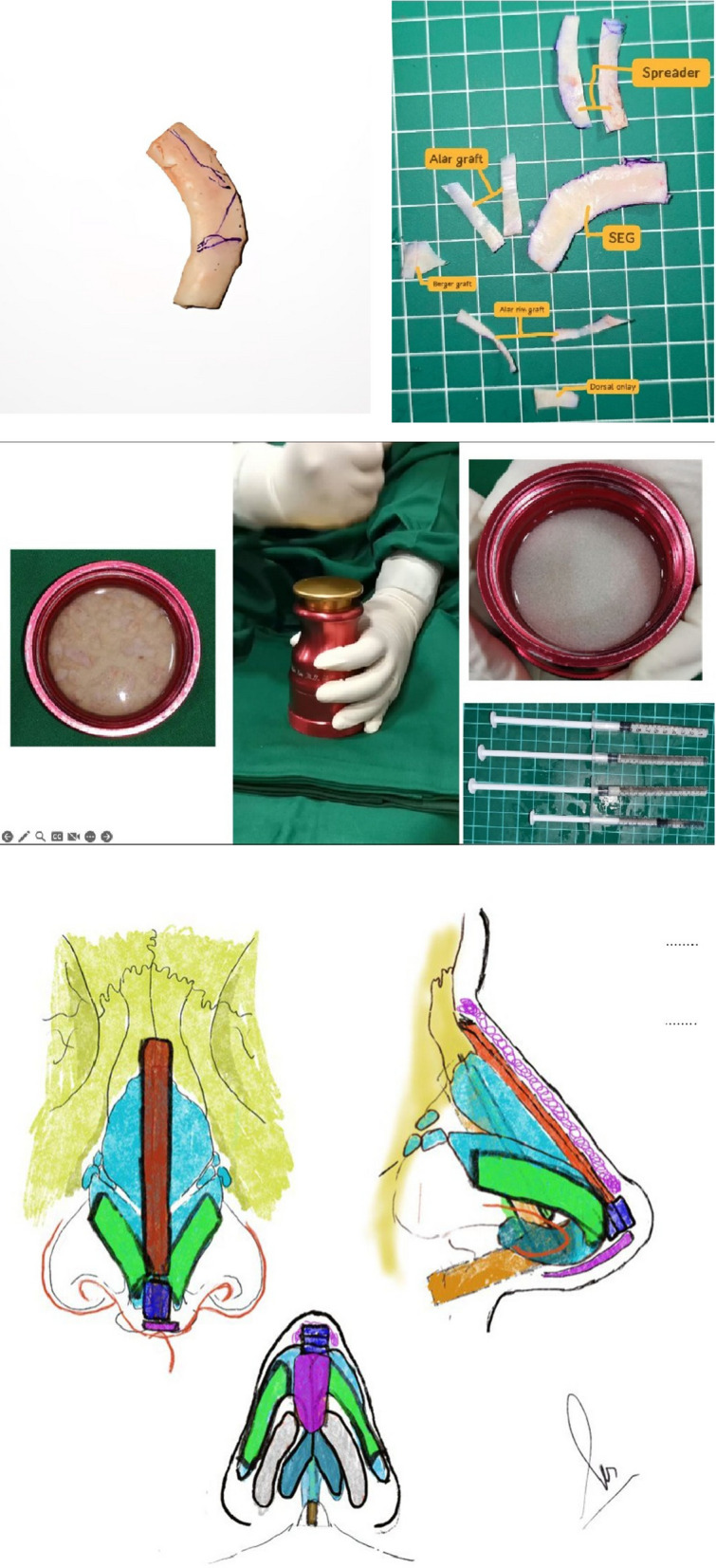
Illustration of key stages in secondary open structural rhinoplasty using autologous costal cartilage grafts. The image shows harvested costal cartilage and sculpted graft components, including septal extension graft, spreader graft, dorsal onlay graft, alar graft, and costal slice, prepared on a sterile grid. The preparation of diced cartilage graft is demonstrated, including the grating, compression, and syringe packing for precise insertion. The schematic diagrams depict the anatomical placement of various graft components—such as septal extension graft, spreader graft, dorsal onlay graft, shield graft, and alar grafts—used to restore nasal structure and symmetry in cleft nasal reconstruction

To address these challenges, various innovative surgical techniques have been introduced, such as upper and/or lower lateral cartilage suspension [11], V–Y chondromucosal composite flaps [12], and cartilage grafting utilizing autologous rib or auricular cartilage [13–15]. To facilitate better understanding and reproducibility of our surgical approach, we have provided schematic illustrations detailing the specific technical steps routinely employed at our institution in secondary open structural rhinoplasty. These include key stages such as costal cartilage harvesting, sculpting of graft components (e.g., septal extension graft, spreader graft, dorsal onlay graft), and precise graft placement techniques used to reconstruct nasal framework in cleft lip and palate patients (see Fig. 1).

Figure 1 schematically illustrates the positions of the various grafts used in our surgical technique, including septal extension grafts, spreader grafts, alar grafts, and shield grafts, providing a concise visual reference aligning with the detailed descriptions in the text.

Vass et al. [16] proposed a standardized approach to secondary septorhinoplasty in CLP patients to achieve more predictable functional and aesthetic outcomes, especially in individuals who may already be affected psychosocially by their condition. Autologous costal cartilage is considered one of the most reliable grafting materials in nasal reconstruction, offering an abundant supply of cartilage with excellent structural support and minimal immunogenic risk due to its autologous origin [17]. Typically, cartilage is harvested from the sixth or seventh rib, while the eighth or ninth ribs may be used when additional volume is required [17, 18]. Furthermore, Ujam et al. [19] reported that the tenth costal cartilage is also a valuable graft source, citing its ease of harvest, dynamic properties, and consistent results with low complication rates. A common concern among patients, particularly females, is the postoperative scar on the chest wall. To minimize aesthetic impact, the incision is ideally placed within the inframammary fold, allowing the scar to be concealed naturally [17, 18].

Autologous costal cartilage has been widely utilized in secondary rhinoplasty for the correction of cleft lip nasal deformities, demonstrating significant postoperative improvements in nasal appearance [20]. An et al. [21] reported that the use of diced costal cartilage grafts in combination with muscle repositioning effectively enhanced the nasal structural base and improved alar symmetry. Similarly, Diao et al. [22] incorporated autologous costal cartilage with fascia grafts, resulting in highly favorable outcomes. Autologous costal cartilage with fascia grafts consistently yields highly favorable outcomes primarily due to its robust structural integrity, versatility, resistance to resorption, and excellent biocompatibility. The fascia component effectively stabilizes diced cartilage, minimizing risks of graft displacement and contour irregularities, while simultaneously promoting smooth and natural contour outcomes. Moreover, this combination reduces graft visibility and palpability, enhancing both functional and aesthetic satisfaction. The mean score on the Independent Rhinoplasty Outcome Score (IROS) indicated a “very good” subjective assessment, reflecting both functional and aesthetic satisfaction.

Therefore, we present a case series of patients who underwent secondary nasal reconstruction using autologous costal cartilage grafts, with the aim of improving both nasal function and aesthetic outcomes. Objective evaluations included measurements of the tip projection ratio and alar width ratio, which were assessed preoperatively and postoperatively to quantify surgical results.

## Case presentation

We conducted a retrospective, single-center, non-consecutive case series involving eight patients diagnosed with cleft lip and palate (CLP) between 2019 and 2024 at a plastic reconstructive and aesthetic surgery unit. The cohort consisted of four male and four female patients. All male patients presented with unilateral CLP, while among the female patients, three had unilateral CLP and one had bilateral CLP. The patients’ ages ranged from 15 to 28 years, with a mean age of 21 years. Objective evaluations included preoperative and postoperative measurements of the tip projection ratio and alar width ratio.

Due to the retrospective nature of this study, photographs were collected from previously documented clinical records. Consequently, standardized camera settings, patient positioning, and lighting conditions were not prospectively established. Although we attempted to maintain consistency in photographic technique across patients, variations were inevitable and represent a limitation of this analysis. We acknowledge that the retrospective nature of our study precluded the establishment of prospective photographic standardization protocols, including camera settings, lighting conditions, and exact patient positioning. This limitation may contribute to measurement variability and potential bias in anthropometric analysis. To assess the reliability of landmark identification in photographic analysis retrospectively, we conducted an inter-rater reliability test using intraclass correlation coefficients (ICC). Two independent raters analyzed all photographic data, resulting in an ICC of 0.89, indicating strong reliability.

Written informed consent was obtained from all patients for the publication of their clinical data and accompanying images. For the 15-year-old patient, written informed consent was obtained from the patient’s parent/guardian. A copy of the signed consent forms is available for review by the Editor of this journal.

### Case 1

**Fig. 2:**
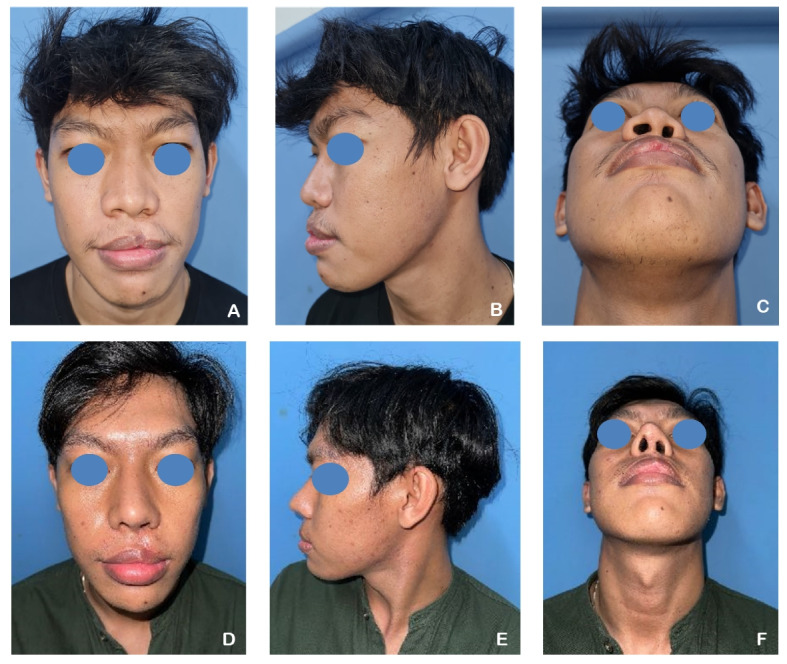
**A**–**C** A 24-year-old male patient with a unilateral complete left cleft lip and palate (CLP) underwent revision labioplasty and full open rhinoplasty. **D**–**F** Six months postoperatively, following reconstruction with autologous rib cartilage grafts. Notable improvements were observed, including an increase in tip projection ratio and a reduction in alar width ratio, contributing to enhanced nasal symmetry and definition

A 24-year-old male patient presented with a unilateral complete left cleft lip and palate (CLP), with a history of labioplasty performed 23 years prior. The patient underwent revision labioplasty in conjunction with full open rhinoplasty. Autologous rib cartilage grafts were utilized and shaped into multiple components, including a septal extension graft, spreader graft, dorsal onlay graft, perichondrium graft, Berger graft, alar graft, shield graft, and diced cartilage graft. Postoperative assessments demonstrated an increase in the tip projection ratio and a decrease in the alar width ratio, indicating improved nasal contour and symmetry (Fig. 2).

### Case 2

**Fig. 3:**
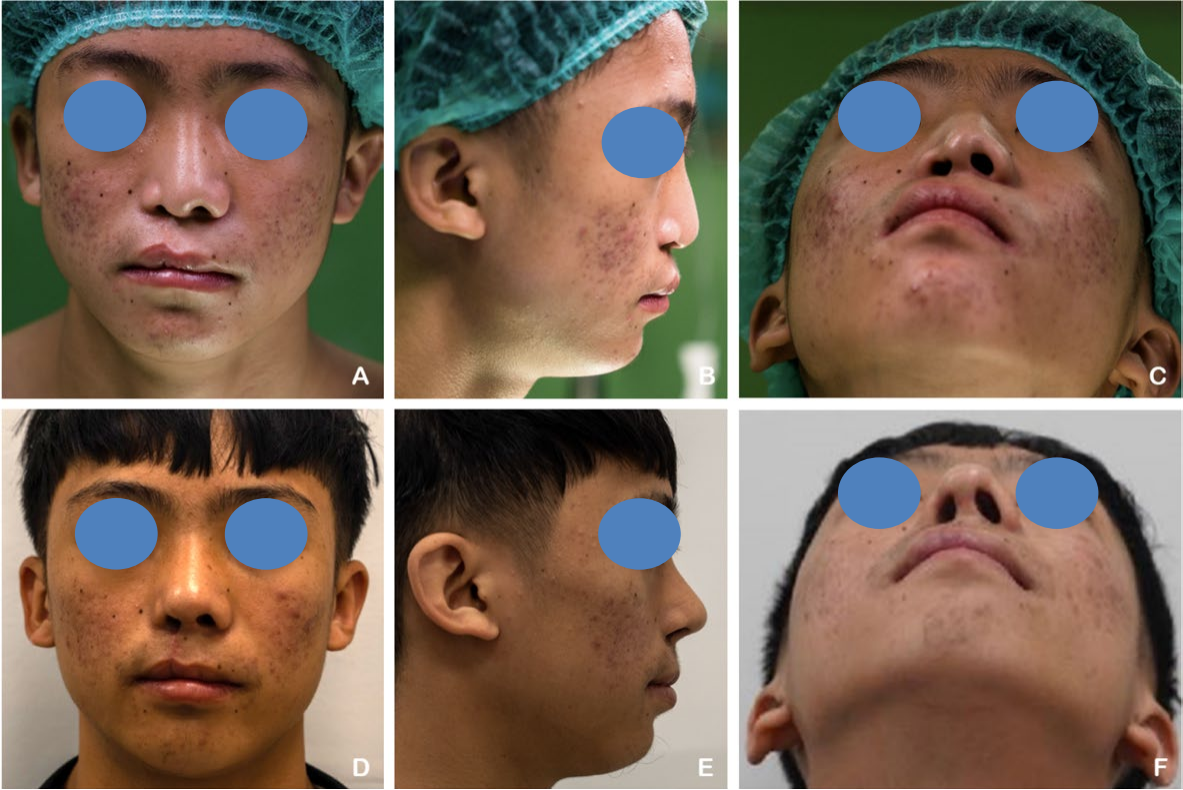
**A**–**C** A 17-year-old male patient with unilateral complete right cleft lip and palate (CLP) underwent revision labioplasty and full open rhinoplasty. **D**–**F** Six months postoperatively, following nasal reconstruction using autologous rib cartilage grafts. Postoperative assessment demonstrated increased tip projection ratio and decreased alar width ratio, reflecting enhanced nasal symmetry and projection.

A 17-year-old male patient with a unilateral complete right cleft lip and palate (CLP) had previously undergone labioplasty 14 years ago and palatoplasty 12 years ago. The patient underwent revision labioplasty combined with full open rhinoplasty. Autologous rib cartilage was harvested and sculpted into a septal extension graft, spreader graft, alar graft, strut graft, alar rim graft, and diced cartilage graft. Postoperative evaluation revealed an increased tip projection ratio and a decreased alar width ratio, indicating improved nasal projection and symmetry (Fig. 3).

### Case 3

**Fig. 4:**
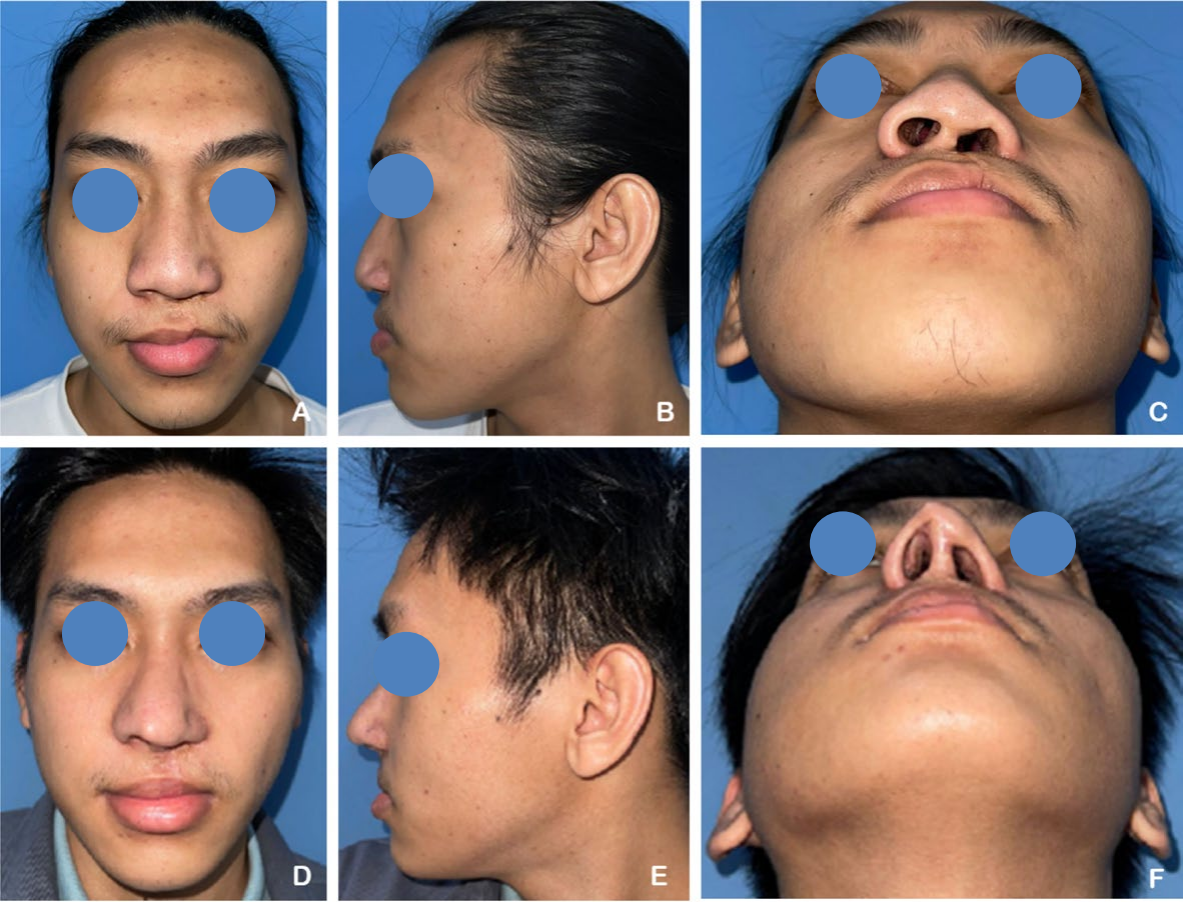
**A–C** A 22-year-old male patient with unilateral complete left cleft lip and palate (CLP) underwent full open rhinoplasty. **D–F** Six months postoperatively, following reconstruction using autologous rib cartilage grafts. The tip projection ratio increased and the alar width ratio decreased, indicating improved nasal projection and symmetry

A 22-year-old male patient with a unilateral complete left cleft lip and palate (CLP) had previously undergone labioplasty 21 years earlier. The patient underwent secondary rhinoplasty utilizing autologous rib cartilage grafts, which were fashioned into a septal extension graft, spreader graft, alar graft, dorsal onlay graft, and diced cartilage graft. Postoperative evaluation revealed an increase in the tip projection ratio and a decrease in the alar width ratio, indicating improved nasal projection and symmetry (Fig. 4).

### Case 4

**Fig. 5:**
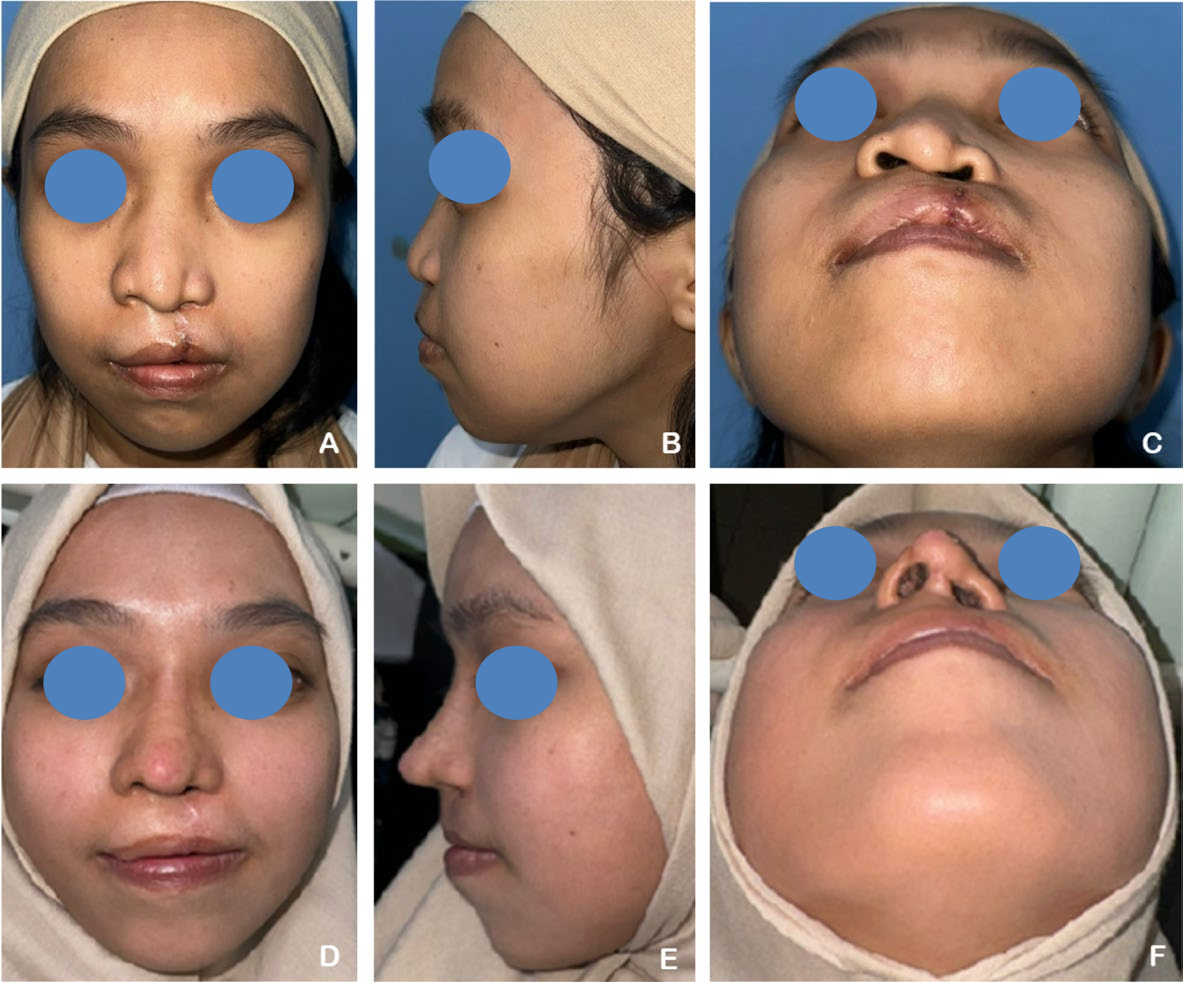
**A–C** A 23-year-old female patient with unilateral complete left cleft lip and palate (CLP) underwent open rhinoplasty. **D–F** Six months postoperatively, following nasal reconstruction using autologous rib cartilage grafts. The tip projection ratio increased and the alar width ratio decreased, indicating improved nasal projection and symmetry

A 23-year-old female patient with unilateral complete left cleft lip and palate (CLP) had previously undergone labioplasty 8 years ago, followed by palatoplasty and revision labioplasty 3 months prior to the current procedure. She underwent open rhinoplasty using autologous rib cartilage grafts, which were shaped into a septal extension graft, spreader graft, alar graft, alar rim graft, Berger graft, shield graft, and diced cartilage graft. Postoperative evaluation demonstrated an increase in the tip projection ratio and a decrease in the alar width ratio, indicating improved nasal projection and contour (Fig. 5).

### Case 5

**Fig. 6:**
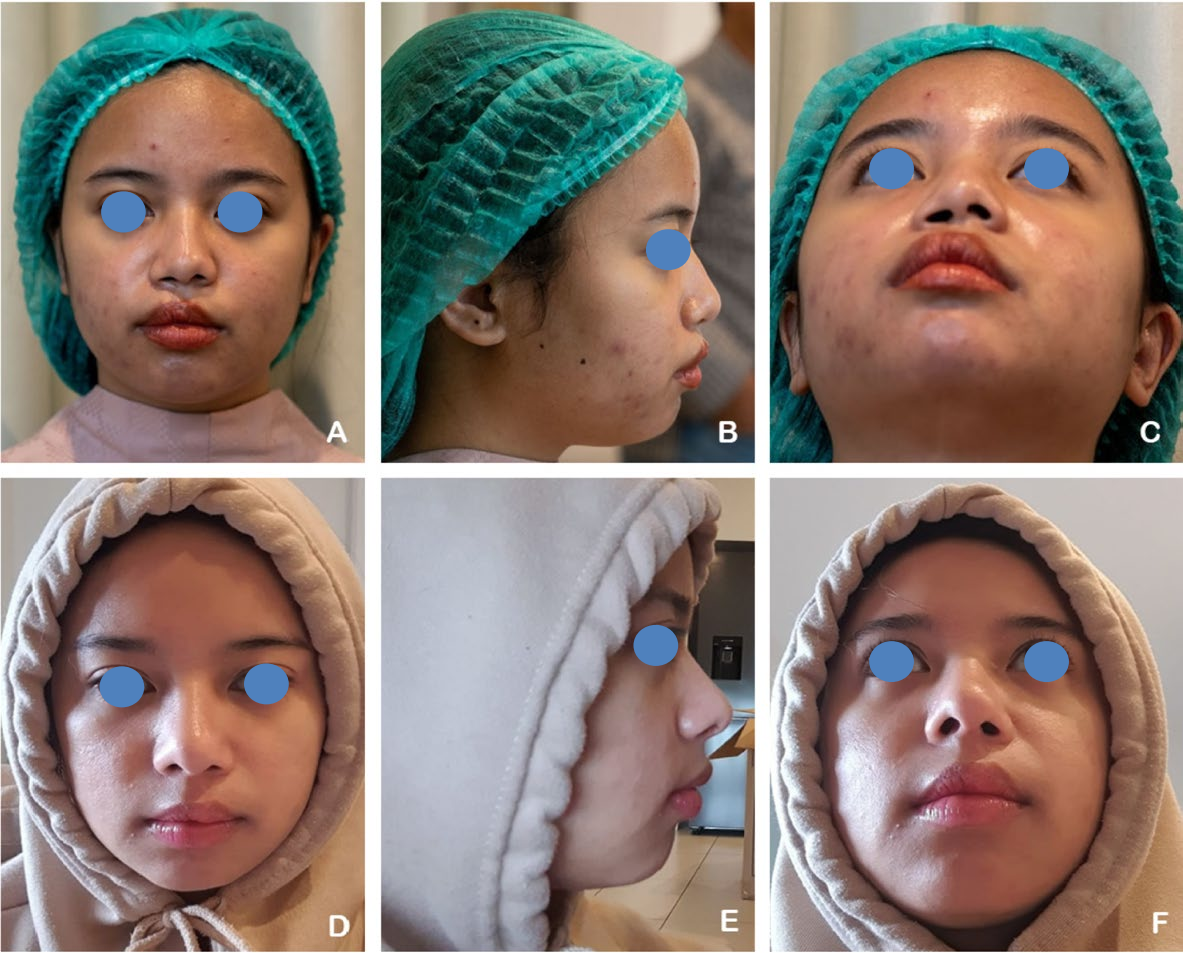
**A–C** A 21-year-old female patient with bilateral asymmetric cleft lip and palate (CLP) underwent open rhinoplasty. **D–F** Six months postoperatively, following reconstruction using autologous rib cartilage grafts. The tip projection ratio increased and the alar width ratio decreased, indicating improved nasal projection and symmetry

A 21-year-old female patient with bilateral asymmetric cleft lip and palate (CLP) had previously undergone labioplasty 20 years ago and palatoplasty 19 years ago. She underwent open rhinoplasty using autologous rib cartilage grafts, which were fashioned into a septal extension graft, spreader graft, alar graft, dorsal onlay graft, Berger graft, and diced cartilage graft. Postoperative evaluation demonstrated an increased tip projection ratio and a decreased alar width ratio, indicating improved nasal projection and symmetry (Fig. 6).

### Case 6

**Fig. 7:**
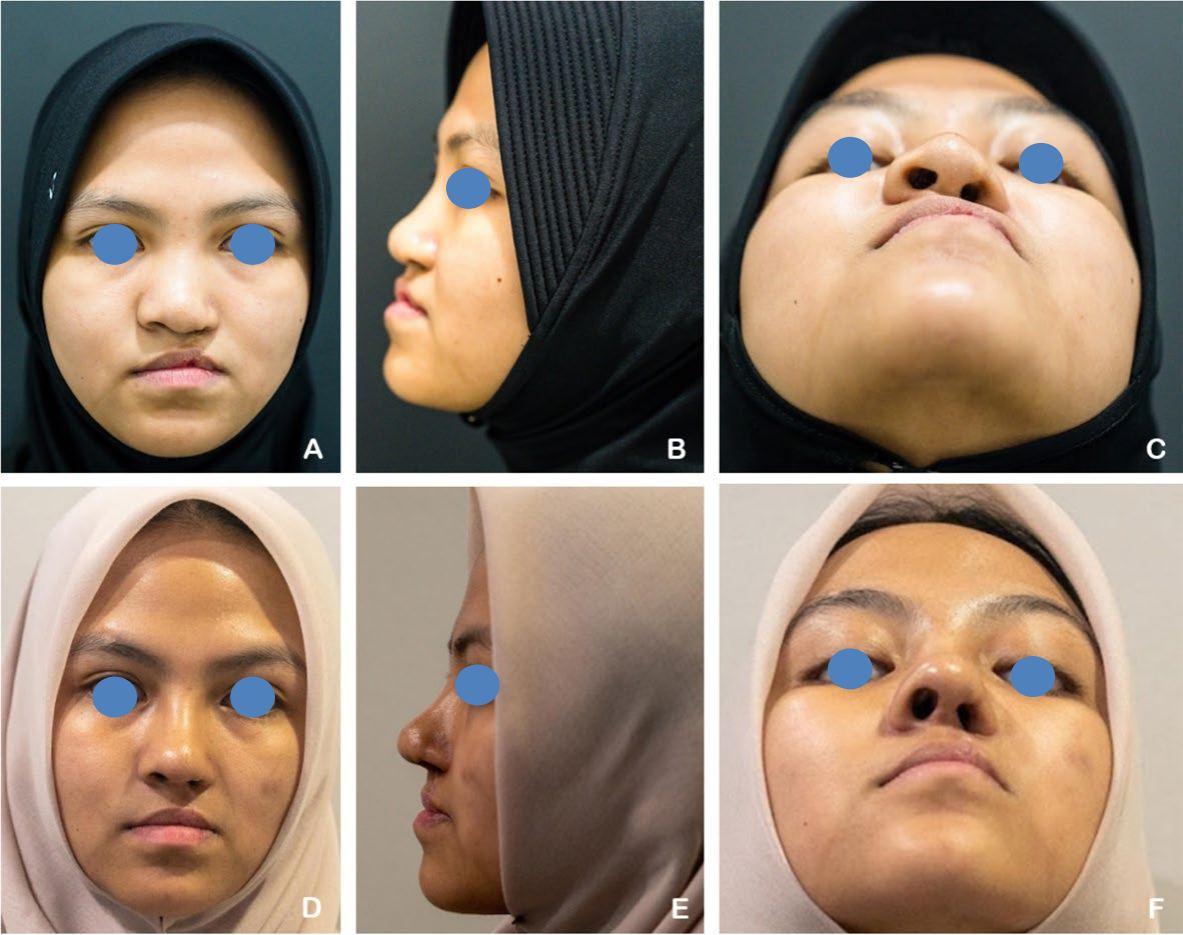
**A**–**C** An 18-year-old female patient with unilateral complete left cleft lip and palate (CLP) underwent revision labioplasty and open rhinoplasty. **D**–**F** Six months postoperatively, following reconstruction using autologous rib cartilage grafts. The tip projection ratio increased and the alar width ratio decreased, indicating enhanced nasal projection and symmetry

An 18-year-old female patient with unilateral complete left cleft lip and palate (CLP) had previously undergone labioplasty 17 years ago and palatoplasty 16 years ago. Revision labioplasty and open rhinoplasty were performed using autologous rib cartilage grafts, which were fashioned into a septal extension graft, spreader graft, dorsal onlay graft, alar graft, Berger graft, and diced cartilage graft. Postoperative evaluation revealed an increase in the tip projection ratio and a decrease in the alar width ratio, indicating improved nasal symmetry and projection (Fig. 7).

### Case 7

**Fig. 8:**
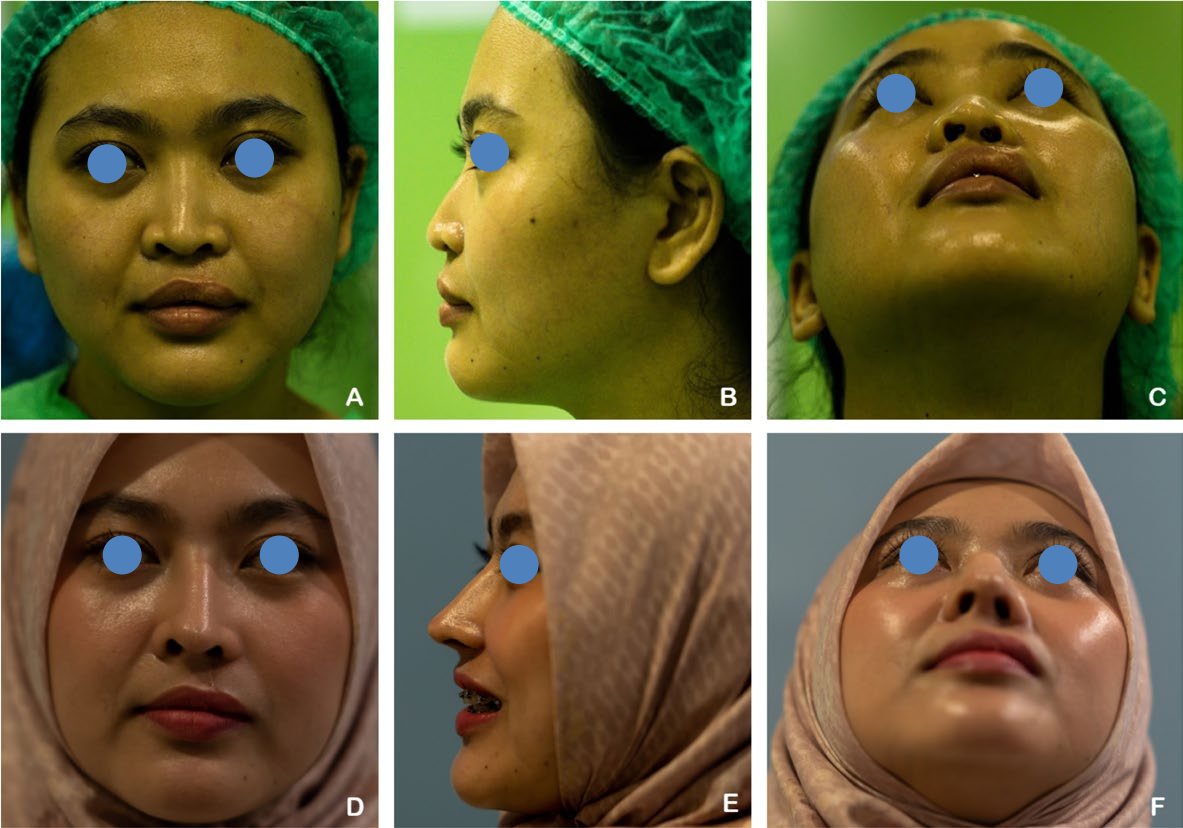
**A**–**C** A 28-year-old female patient with unilateral left cleft lip and palate (CLP) underwent revision labioplasty and open rhinoplasty. **D**–**F** Six months postoperatively, following nasal reconstruction using autologous rib cartilage grafts. The tip projection ratio increased and the alar width ratio decreased, reflecting improved nasal projection and symmetry

A 28-year-old female patient with unilateral left cleft lip and palate (CLP) had previously undergone labioplasty 27 years ago and palatoplasty 26 years ago. She underwent revision labioplasty and open rhinoplasty using autologous rib cartilage grafts, which were shaped into a septal extension graft, spreader graft, alar graft, alar rim graft, Berger graft, dorsal onlay graft, and diced cartilage graft. Postoperative evaluation demonstrated an increased tip projection ratio and a decreased alar width ratio, reflecting improved nasal projection and symmetry (Fig. 8).

### Case 8

A 15-year-old male patient with unilateral right cleft lip and palate (CLP) had previously undergone labioplasty 14 years ago and palatoplasty 13 years ago. He underwent open rhinoplasty using autologous rib cartilage grafts, which were fashioned into a septal extension graft, spreader graft, dorsal onlay graft, alar graft, alar rim graft, and diced cartilage graft. Postoperative evaluation revealed an increased tip projection ratio and a decreased alar width ratio, indicating enhanced nasal projection and symmetry 

**Fig. 9:**
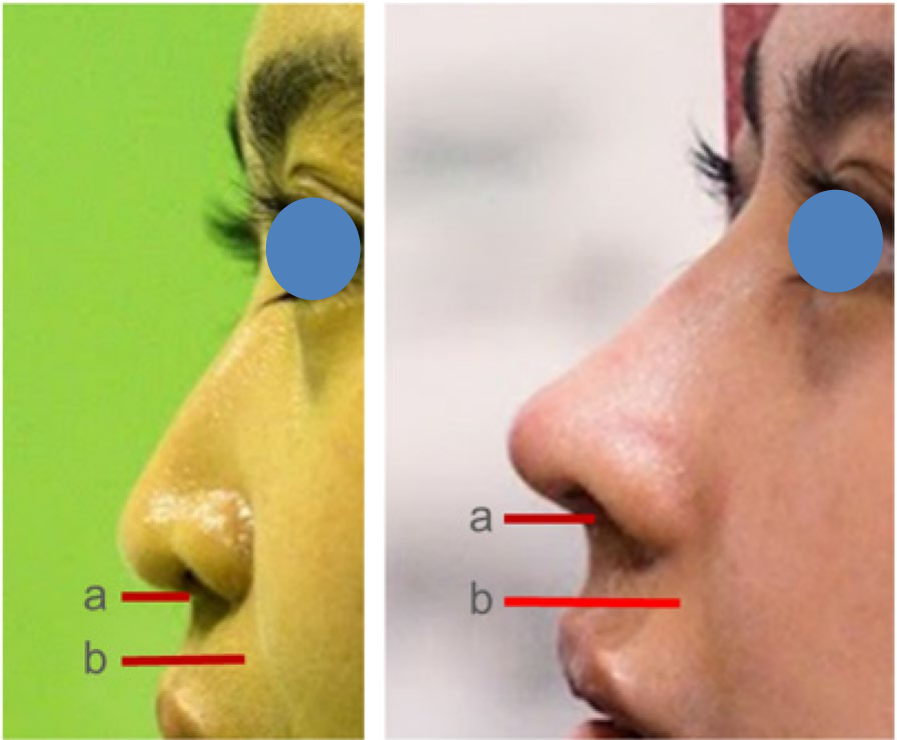
Measurement of tip projection ration. **a** Tip. **b** Nasal base

**Fig. 10:**
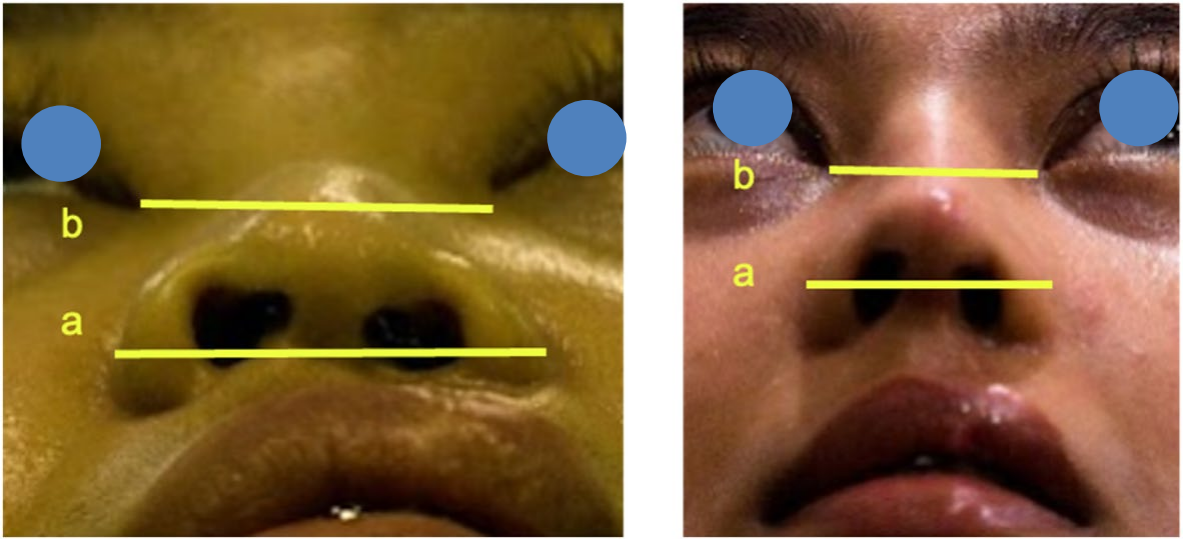
Measurement of alar width ratio. **a** Alar width. **b** Canthal distance

All patients were followed up for 6 months postoperatively. Indirect photographic evaluations were conducted to compare preoperative and postoperative outcomes. Table 1 summarizes the detailed demographic data, previous surgical history, specific graft techniques utilized, and objective anthropometric outcomes (tip projection and alar width ratios) for each patient included in this case series. No complications were recorded during the 6-month postoperative follow-up period. The measurements demonstrated an increase in the tip projection ratio and a decrease in the alar width ratio (Table 2). None of the patients reported complaints regarding nasal appearance, lip contour or shape, or postoperative irregularities (Figs. 9 and 10).

**Table 1:** Detailed individual patient characteristics, surgical history, surgical procedures, outcomes, and complications

Case	Age at surgery (years)	Gender	Type of cleft deformity	Previous surgical history	Surgical procedures (grafts used)	Pre-op tip projection ratio	Post-op tip projection ratio	Pre-op alar width ratio	Post-op alar width ratio	Complication
1	24	Male	Unilateral left CLP	Labioplasty (23 years prior)	Septal extension, spreader, dorsal onlay, perichondrium, Berger, alar, shield, diced cartilage	0.55	0.65	1.18	0.96	None
2	17	Male	Unilateral right CLP	Labioplasty (14 years prior), Palatoplasty (12 years prior)	Septal extension, spreader, alar, strut, alar rim, diced cartilage	0.56	0.67	1.20	0.97	None
3	22	Male	Unilateral left CLP	Labioplasty (21 years prior)	Septal extension, spreader, alar, dorsal onlay, diced cartilage	0.55	0.66	1.17	0.95	None
4	23	Female	Unilateral left CLP	Labioplasty (8 years prior), Palatoplasty and Revision labioplasty (3 months prior)	Septal extension, spreader, alar, alar rim, Berger, shield, diced cartilage	0.55	0.66	1.18	0.95	None
5	21	Female	Bilateral asymmetric CLP	Labioplasty (20 years prior), Palatoplasty (19 years prior)	Septal extension, spreader, alar, dorsal onlay, Berger, diced cartilage	0.56	0.66	1.19	0.97	None
6	18	Female	Unilateral left CLP	Labioplasty (17 years prior), Palatoplasty (16 years prior)	Septal extension, spreader, dorsal onlay, alar, Berger, diced cartilage	0.55	0.64	1.16	0.94	None
7	28	Female	Unilateral left CLP	Labioplasty (27 years prior), Palatoplasty (26 years prior)	Septal extension, spreader, alar, alar rim, Berger, dorsal onlay, diced cartilage	0.55	0.65	1.19	0.96	None
8	15	Male	Unilateral right CLP	Labioplasty (14 years prior), Palatoplasty (13 years prior)	Septal extension, spreader, dorsal onlay, alar, alar rim, diced cartilage	0.56	0.66	1.18	0.95	None

**Table 2:** Preoperative and postoperative measurement of tip projection ratio and alar width ratio

Measurement	Preoperative (*n* = 8)	Postoperative (*n* = 8)	Independent *t* test *p*-value (significant *p* < 0.05)	Ratio changes
Tip projection ratio (Tip [a]/nasal base [b])	0.554	0.65	0.03	0.12
Alar width ratio (Alar width [a]/canthal distance [b])	1.18	0.96	0.035	− 0.17

*Tip (a)* defined as the distance from the alar-facial groove to the nasal tip apex in lateral view photographs

*Nasal base (b)* defined as the horizontal distance between the alar-facial grooves measured from the frontal view photographs

Although substantial improvements in nasal symmetry were achieved, slight residual nostril asymmetry remained evident in some cases, reflecting inherent challenges in completely correcting complex nasal deformities associated with cleft lip and palate. This highlights the necessity for realistic patient expectations regarding achievable symmetry.

## Discussion

Reconstruction of cleft lip and palate (CLP) involves correction of both soft tissue and skeletal deformities. In unilateral CLP, characteristic nasal features include a deviated nasal tip, unilaterally shortened columella, elongated lateral crus, shortened medial crus, blunted dome, and poorly defined nasal tip. The alar base on the cleft side is typically displaced posteriorly, laterally, and inferiorly. Additional findings often include a widened alar base, an absent or depressed nasal floor, and deviation of the septum and anterior nasal spine toward the non-cleft side.

In bilateral CLP, the orbicularis oris muscle is absent in the prolabium. Patients typically present with a broad nasal tip, shortened and retracted columella, absent nasal floor, disfigured medial crura, a low nasal dome, and deviated caudal septum. Soft tissue anomalies frequently include a deviated columella, short columellar skin, short alar mucosa, redundant soft triangle tissue, and a depressed nasal seal [13].

A short columella is a major challenge in both unilateral and bilateral CLP. Postoperative scar contracture and limited available tissue complicate efforts to achieve satisfactory nasal symmetry. Several surgical techniques have been proposed to address these complex deformities. Hafezi et al. described an effective approach using autologous rib cartilage fashioned into spreader and inlay grafts, achieving acceptable aesthetic outcomes [13]. An et al. utilized diced costal cartilage in combination with spreader and columellar strut grafts to address alar asymmetry in secondary cleft rhinoplasty [21].

In our series, secondary rhinoplasty was performed using autologous rib cartilage, sculpted into various graft types tailored to each patient’s specific anatomical deformities. These included septal extension grafts, spreader grafts, dorsal onlay grafts, perichondrium grafts, Berger grafts, alar and alar rim grafts, shield grafts, and diced cartilage grafts.

Spreader grafts were carved either from resected septal cartilage or rib cartilage segments. These grafts were positioned between the septum and upper lateral cartilages to reconstruct the internal nasal valve and maintain midline alignment of the septum [10]. Septal extension grafts were applied to enhance nasal tip support and projection [23]. When used, columellar strut grafts were anchored between the domes of the lower lateral cartilages and the caudal septum, and secured to adjacent spreader grafts. Lateral crus reconstruction involved batten or strut grafts placed either beneath the residual crus or in an onlay configuration. Shield grafts were employed to further refine tip symmetry, projection, and shape [10].

Secondary rhinoplasty provides both aesthetic and functional improvements in CLP patients. Multiple authors advocate the use of autologous rib cartilage as the ideal grafting material in these cases [24–27]. Indications include severe nasal projection deficiency, inadequate septal cartilage, and the need for large cartilage volumes. Rib cartilage offers strong structural support, resists scar contracture, and is readily available in sufficient quantity [10]. In our series, sixth-rib cartilage was used consistently to ensure safety and compatibility due to its autologous nature [17].

In comparison to recent studies, our postoperative outcomes showed similar or slightly superior improvements in nasal tip projection and alar width reduction. For instance, An et al. (2021) reported an average increase in nasal tip projection ratios ranging from 0.08 to 0.12, closely aligning with our result of 0.11. Similarly, Diao et al. (2024) reported favorable results regarding symmetry improvements using costal cartilage and fascia grafts, highlighting consistent patient satisfaction and minimal complication rates. Our series further validates these findings by demonstrating no complications across all cases.

The specific selection of graft types, notably the consistent use of Berger grafts, diced cartilage grafts, and shield grafts, differentiates our technique from others described in the literature. While spreader and septal extension grafts are commonly used, our systematic combination with these additional graft types effectively addresses individual anatomical variations, enhancing overall nasal symmetry and projection.

Regarding patient satisfaction, our subjective assessments indicate high satisfaction, paralleling findings reported by Cervelli et al. (2006) and Yilmaz et al. (2007), who noted significant aesthetic improvements and psychological benefits postoperatively.

Despite its advantages, the use of rib cartilage is not without challenges. Potential issues include the need for additional grafts, risk of pneumothorax, postoperative pain, warping, and visible chest wall scarring [18]. In our study, none of the patients required supplemental grafts, and no instances of pneumothorax or cartilage warping were observed during follow-up. Postoperative pain was adequately managed in all cases.

Cartilage calcification is another potential limitation, particularly in older patients, and may increase the risk of warping. Preoperative magnetic resonance imaging (MRI) can assist in evaluating the degree of calcification. If calcification is minimal and sculpting remains feasible, warping can be minimized or avoided altogether [28].

## Conclusion

Reconstruction of cleft lip and palate–associated nasal deformities remains one of the most complex challenges in facial plastic surgery. Secondary structural rhinoplasty using autologous rib cartilage grafts is considered a definitive treatment modality for addressing these deformities. The primary objectives include achieving nasal symmetry, restoring function, and improving tip projection. A systematic and carefully planned approach is essential to reconstruct key anatomical components, including the septum, internal nasal valve, nasal tip, alar contours, alar base symmetry, columellar length, and dorsal profile. In this study, postoperative anthropometric evaluations demonstrated significant improvements, particularly in nasal tip projection and alar base narrowing. No major intraoperative or postoperative complications were encountered.

## Data Availability

No datasets were generated or analysed during the current study. The authors declare no competing interests.

## Abbreviations

CLP: Cleft lip and palate
CN: Cleft nasal
IROS: Independent Rhinoplasty Outcome Score
MRI: Magnetic resonance imaging
IRB: Institutional Review Board
